# A new clinical decision support tool for improving the adequacy of anticoagulant therapy and reducing the incidence of stroke in nonvalvular atrial fibrillation: A randomized clinical trial in primary care

**DOI:** 10.1097/MD.0000000000009578

**Published:** 2018-01-19

**Authors:** Maria Rosa Dalmau Llorca, Alessandra Queiroga Gonçalves, Emma Forcadell Drago, José Fernández-Sáez, Zojaina Hernández Rojas, Josep Maria Pepió Vilaubí, Dolores Rodríguez Cumplido, Rosa Maria Morral Parente, Carina Aguilar Martín

**Affiliations:** a Equip d’Atenció Primària Tortosa Oest, Institut Català de la Salut; b Unitat de Suport a la Recerca Terres de l’Ebre, Institut Universitari d’Investigació en Atenció Primària (IDIAP) Jordi Gol; c Unitat Docent de Medicina de Família i Comunitària Tortosa-Terres de L‘Ebre, Institut Català de la Salut, Tortosa; d Universitat Autònoma de Barcelona, Bellaterra, Cerdanyola del Vallès; e Equip d’Atenció Primària Ulldecona-La Sénia; f Equip d’Atenció Primària Tortosa Est, Institut Català de la Salut, Tortosa; g Fundació Institut Català de Farmacologia, Universitat Autònoma de Barcelona; h Direcció Assistencial d’Atenció Primària, Institut Català de la Salut, Barcelona; i Unitat d’Avaluació, Direcció d’Atenció Primària Terres de l’Ebre, Institut Català de la Salut, Tortosa, Tarragona, Spain.

**Keywords:** anticoagulants, atrial fibrillation, clinical decision support systems, incidence, primary health care, randomized controlled trial, stroke

## Abstract

**Introduction:**

Atrial fibrillation (AF) is the most common cardiac arrhythmia and increases the risk of ischemic stroke 4 to 5-fold. The first choice of anticoagulant therapy (AT) is the vitamin K antagonist (VKA). Contraindication to VKA or poor control of the International Normalized Ratio leads to the administration of direct-acting oral anticoagulants. There is a trend toward inadequate AT in nonvalvular AF (NVAF) patients. Aim: To evaluate the impact of the implementation of a decision support tool linked to the digital clinical history on the adequacy of AT, the incidence of complications, and the mortality in patients with NVAF in primary care centers (PCCs) of the Catalan Institute of Health (ICS).

**Methods and analysis:**

Randomized clinical trial in 287 PCCs, formed by 2 groups (intervention and control). Population: patients controlled in PCCs, diagnosed with NVAF 1 year before the implementation of the decision support tool and with VKA treatment over a minimum of 1 year. A simple randomization method will be performed at a sector level. The decision support tool will be available for 1 year. The time in therapeutic range (TTR) will be available in the digital clinical history only to professionals of the intervention group. The information system for primary care research development database will be used for the data extraction. Statistical analysis will be done at 3 time points: before the implementation of the tool, at 1 year, and at 2 years after the beginning of the intervention. Multilevel (patient and professional levels) logistic regression models will be used to estimate the effect of the intervention.

**Ethics and dissemination:**

This study protocol was approved by the Ethical Committee of Clinical Investigation of the *Institut Universitari d’Investigació en Atenció Primària Jordi Gol* (code P17/091). Articles will be published in scientific journals.

**Trial registration:**

Clinical-Trials.gov: NCT03367325.

## 1 Introduction

Atrial fibrillation (AF) is the most frequent type of cardiac arrhythmia, and patients with AF have a 4 to 5-fold greater risk of ischemic stroke than the general population.^[1,2]^ This arrhythmia affects more than 6 million people in the European Union, with an estimated prevalence of 1.5% to 2% in the general population, increasing to up to 24.4% in individuals of more than 85 years of age.^[1,3–5]^

According to the European Heart Rhythm Association, nonvalvular AF (NVAF) is produced in the absence of mechanical prosthetic heart valves and the absence of moderate to severe mitral stenosis.^[6]^ NVAF carries an increased risk of death, morbidity, and hospitalization with the subsequent increase in healthcare costs.^[1,7–9]^ Although the physiopathology of NVAF is well-known, the diagnosis and management of these patients needs to be improved.^[10]^ The main objective of the management of these patients is to reduce the symptoms and prevent the severe complications associated with this arrhthymia. The prevention of thromboembolic complications is based on antithrombotic treatment with either oral anticoagulants or antiplatelet drugs.^[1,8]^

Vitamin K antagonists (VKAs) were the only oral anticoagulants available for many years. However, the characteristics of VKAs may make their use in patients with NVAF challenging in clinical practice, leading to difficulties in the management of these patients and the need to adapt the therapy.^[11]^ Recently, direct-acting oral anticoagulants (DOACs) were developed and have shown to be safe and effective in the prevention of stroke and systemic embolism in patients with NVAF, although some limitations have also been reported.^[12]^

Since the introduction of DOACs in Spain their use has been on the rise. Nevertheless, data from the Catalan Health Service on the adequacy of DOAC prescriptions indicate that according to the recommendations of the pharmacotherapy commission more than 25% of the patients are not candidates for dabigatran treatment and are receiving it, whereas patients who are candidates to this treatment are not receiving the drug.^[13]^ These results indicate a clear problem of adequacy to the anticoagulant treatment. We believe that this problem is probably because of the absence of any single variable in the electronic clinical history of the Catalan Institute of Health (ICS) that provides direct knowledge regarding control of the international normalized ratio (INR), thereby making it difficult to know the real level of control in the previous 6 months, and consequently, the adequacy of treatment and the possible changes needed.

The present project has been designed taking into account the above situation. The aim of the project is to improve the information available in the electronic clinical history by direct calculation of the time in the therapeutic range (TTR) once the value of the last INR of NVAF patients receiving VKA has been entered. When the TRT is < 65% at 6 months, an alert will be activated and will inform the professional of the need to evaluate a change in the treatment. The TTR (calculated using the Rosendaal method^[14]^) is a widely applied measure for the evaluation of the adequacy of anticoagulant therapy (AT) with VKAs. In several guidelines on the management of AF the TTR is used as a treatment control criterion, although the use of these guidelines are not generalized throughout the world.^[15]^ According to the recommendations of the ministry of health, social services and equality of Spain (based on the National Institute for Health and Care Excellence guideline^[16]^) for the use of DOAC in NVAF patients, a TTR <65% is a criterion of poor VKA control.^[11]^

Despite the increasingly more frequent use of computerized medical reminders, few studies have assessed their impact on health outcomes.^[17–21]^ A systematic review showed that notifications influence specific aspects of the prescription process and may be an effective method to reduce the prescription of contraindicated medications, although their impact on health outcomes was not demonstrated.^[22]^ Within the primary care setting it was found that computerized clinical notifications significantly improved the adequacy of treatment in patients with poorly controlled asthma and consequently reduced the rate of reexacerbations during follow-up. Moreover, medical professionals considered these reminders to be particularly useful for the identification of patients with bad control.^[23]^

The present project aims to evaluate the impact of the implementation of a clinical decision support tool associated with the electronic clinical history which informs healthcare professionals of the TTR in patients with NVAF attended in primary care centers (PCCs) of the ICS. This project will evaluate the impact of this tool on the adequacy of anticoagulant treatment, the incidence of thromboembolic and hemorrhagic complications, and mortality at 2 years after the beginning of the intervention. The evaluation of the impact of the intervention will be performed based on the characteristics of the patients and the healthcare professionals.

## 2 Methods and design

### 2.1 Study design and setting

A cluster, randomized, clinical trial (type: parallel group) will be performed with patients attended in the ICS primary care setting. It is estimated that 5.8 million people are attended in ICS which comprises 287 PCCs around Catalunya (source: Sistemes d’Informació dels Serveis d’Atenció Primària (SISAP) of the ICS, unpublished data).

### 2.2 Participants

The study will be carried out in patients diagnosed with NVAF receiving anticoagulant treatment.

#### 2.2.1 Inclusion criteria (all criteria must be met)

- Patients diagnosed with NVAF 1 year prior to the implementation of the computerized tool;
- Patients receiving anticoagulant treatment with DOACs or VKAs;
- Patients followed in primary care (with at least 6 INR controls during the year prior to the intervention). The restriction related to the INR controls is to minimize the variability in INR at the initiation of treatment and avoid the effect that temporary withdrawal of VKA anticoagulants might have on the percentage of good INR control.

#### 2.2.2 Exclusion criteria

- Patients with INR control in the reference hospital;
- Patients with valvular AF (mitral stenosis);
- Patients with a prosthetic heart valve;
- Change to another PCC.

### 2.3 Intervention

The clinical decision support tool for improving the adequacy of AT in NVAF (CDS-NVAF) was developed by the ICS in collaboration with primary care physicians in 2017. CDS-NVAF is intended for patients diagnosed with NVAF and treated with VKA. This tool calculates the TTR using the Rosendaal method considering the last INR data. The INR data are found in the clinical history of NVAF patients and are located on the screen of the follow-up of oral anticoagulant use.

The CDS-NVAF will be activated when the physician introduces the last INR value. At this time the TTR will be calculated automatically. If the TTR value is <65%, a pop up screen with an advisory text will open suggesting a change to DOAC therapy. The physician can decide whether to change or not the previous prescription. If the TTR value in >65% the pop up screen will not appear. The TTR value will remain registered on the screen of the follow-up of oral anticoagulant use and can be consulted in future queries.

The diffusion of the CDS-NVAF will be made by an announcement on the first day of the intervention when the healthcare professional opens the electronic clinical history. The professionals of the intervention group will receive the announcement and will be provided with the CDS-NVAF. The professionals who are part of the control group will not receive any of the above described information.

The implementation of the CDS-NVAF, and thus, the beginning of the study is expected to December 15, 2017.

### 2.4 Sample size

In a first approach the information system for primary care research development (SIDIAP) established that during 2015 PCCs of the ICS had registered 171,472 cases of NVAF, 73,632 of whom were receiving anticoagulant treatment (63,001 VKAs and 10,631 DOACs). Assuming a weight of 90%, an alpha error of 5%, a control versus intervention ratio of 1, corrected for the design by clusters and assuming a loss of 10%, our study will be able to detect a difference of 1% for a rate of thromboembolic events of 0.66%. Despite this power, a clinically relevant relative reduction of 10% will be established.

### 2.5 Randomization

**Figure 1:**
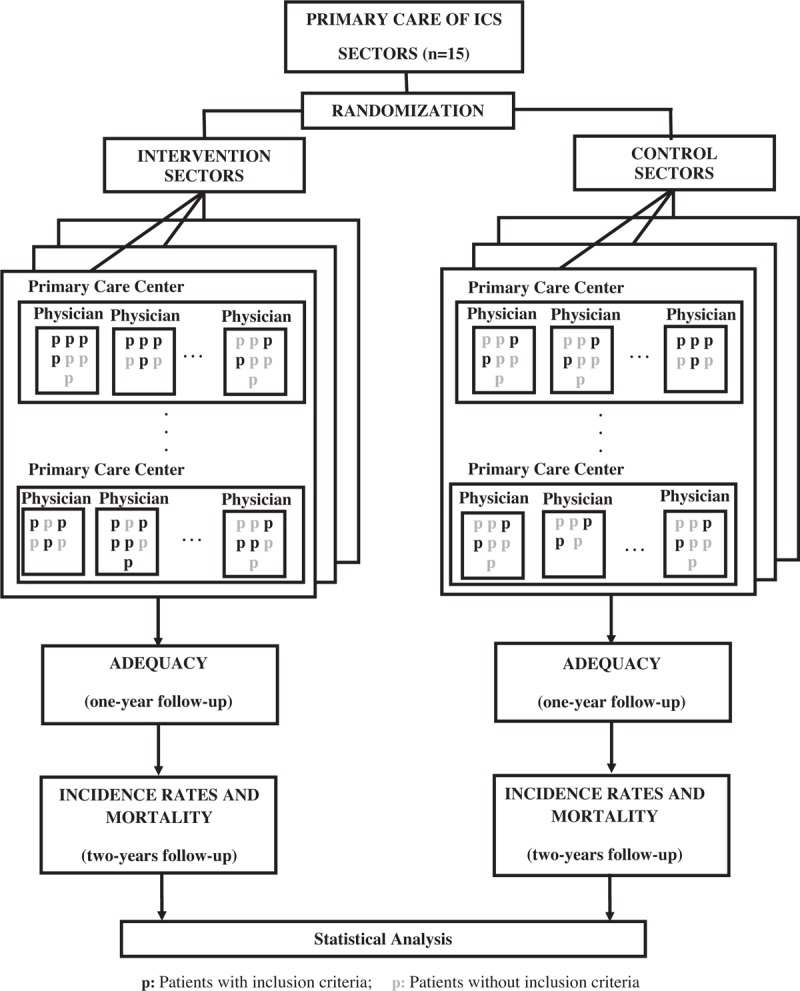
Study flowchart. ICS = Catalan Institute of Health.

The randomization will be made by sectors of the ICS. A sector is a group of PCCs that share 1 computer server. The ICS has 15 sectors. The sectors will be randomized using a simple randomization method. From the generation of random numbers (between 0 and 1) 2 groups will be created so that the numbers with which the first decimal is <5 will be rounded down to 0 and the remaining will be rounded up to 1. The sectors associated with 0 will constitute the control group and those associated with 1 will make up the intervention group. The study flowchart is presented in Figure 1.

### 2.6 Masking

The study will be blind for the trial participants, investigators of the project and data managers, but will not be blind to the physicians.

### 2.7 Data collection

The study variables will be collected from the SIDIAP population database. The SIDIAP has anonymous data of 5.8 million patients in the electronic medical registry system of the ICS and complementary sources. This database includes the following information: sociodemographic variables, healthcare activity, and clinical variables. Pharmacy registries: Information on the medications provided by pharmacies and the associated cost. Laboratory tests. The anonymized data will be exported from the SIDIAP to the statistical package used in the study.

### 2.8 Outcomes

The outcomes of the study are as described below. Health events will be classified according to the International Classification of Diseases-10 code. A schedule for data collection is presented in Table 1.

**Table 1:**
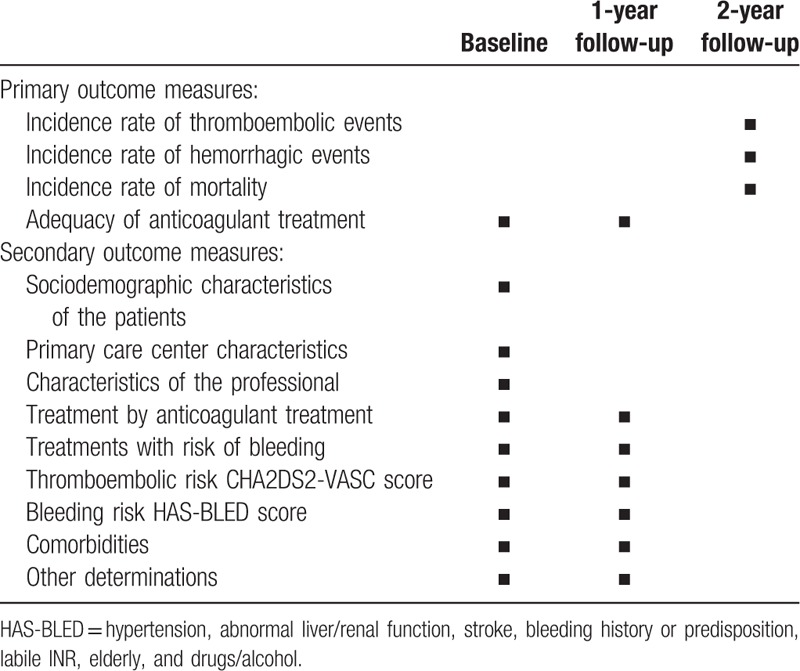
Schedule for data collection.

#### 2.8.1 Primary outcomes

(1) Incidence rate of thromboembolic events (acute myocardial infarction or angina, ischemic stroke, peripheral embolism, or transient ischemic attack [TIA]).
(2) Incidence rates: Hemorrhagic events (intracranial hemorrhage, gastrointestinal hemorrhage); All-cause mortality.
(3) Adequacy of anticoagulant treatment: This variable encompasses the adequacy of the anticoagulant treatment, taking into account if an adequate change has occurred or not. The adequacy of the AT in patients with NVAF will be based on the fulfillment of the criteria of the ministry of health, social services and equality of Spain, 2016^[11]^ (Table 2).

**Table 2:**
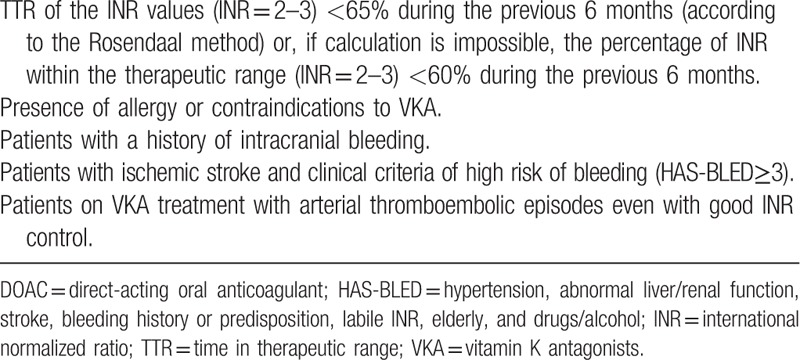
Criteria on the adequacy of treatment with DOACs, according to ministry of health, social services and equality from Spain (2016).

0 = No adequate change (when an inadequate change has been produced or when an inadequate treatment has been maintained).

1 = adequate change (when an adequate change has been produced or when an adequate treatment has been maintained).

#### 2.8.2 Secondary outcomes

(1) Sociodemographic characteristics of the patients: age, sex, primary care area assigned, physician assigned;
(2) PCC characteristics: teaching center, urban/rural, socioeconomic deprivation index (MEDEA), standard of healthcare quality (SHCQ) and the standard of quality of pharmaceutical prescription (SQPP);
(3) Characteristics of the professional: age, sex, PCC, type of work contract, SHCQ, SQPP;
(4) Type of anticoagulant treatment: DOAC (dabigatran, apixaban or rivaroxaban), VKA (acenocoumarol or warfarin), heparin (with starting and completion date);
(5) Type of treatment with risk of bleeding: antiaggregants, nonsteroidal anti-inflammatories, and selective serotonin reuptake inhibitors (with starting and completion date);
(6) Thromboembolic risk CHA2DS2-VASC (congestive heart failure, hypertension, age 75 years or older, diabetes mellitus, previous stroke or TIA, vascular disease, age 65–74 years, female) score (with date of determination). The score indicates the risk of a patient with NVAF of suffering a stroke in 1 year. The score goes from 0 (absence of risk) to 10 (greater risk);
(7) Bleeding risk HAS-BLED (hypertension, abnormal liver/renal function, stroke, bleeding history or predisposition, labile INR, elderly (>65 years), and drugs/alcohol) score (with date of determination). The score allows the calculation of the risk of bleeding in patients with NVAF receiving oral anticoagulant treatment based on the risk factors associated with the probability of bleeding. Score: 0 = low risk; 1 = intermediate risk; 2 = intermediate risk; ≥3 = high risk;
(8) Associated comorbidity (with date of diagnosis): diabetes mellitus, arterial hypertension, heart failure, chronic kidney failure, nephropathy, abnormal liver function, alcoholism, peripheral vascular disease, stroke, peripheral embolism, or TIA;
(9) Other determinations (with date of determination): creatinine, systolic blood pressure, and ejection fraction.

### 2.9 Pilot study

To evaluate the initial functioning of the CDS-NVAF and to promote technical improvements before the beginning of the study, a pilot study was carried out for 15 days (from July 20, 2017 to August 3, 2017). The pilot study consisted in the evaluation of 3 PCCs, 2 of which received the alert in the electronic clinical history with the characteristics described in the ‘intervention’ section.

During the period of the pilot study, aspects about the functionality of the CDS-NVAF and suggestions for improvements were collected from the research team and the primary care professionals (physicians, nurses, and referent professionals) in work meetings. Some changes and suggestions for improvements on the part of professionals were incorporated to improve the functionality of the CDS-NVAF.

### 2.10 Statistical analysis

The statistical analysis will be performed at 3 time points: before the implementation of the CDS-NVAF (baseline), at 1 year after the beginning of the intervention (1-year follow-up), and at 2 years after the beginning of the intervention (2-year follow-up).

A descriptive analysis of all the variables studied will be carried out using frequency and percentage for categorical variables and mean, standard deviation, minimum, median, and maximum for continuous variables. The Students’ *t* test (or the Mann–Whitney U test in cases not fulfilling the criteria of normality) will be used to determine possible differences in the means of the quantitative variables between the 2 study groups. The Chi-square test (or Fisher exact test) will be used for the qualitative variables. The rates of incidence for each variable will be expressed as patient/years with the corresponding confidence intervals of 95%.

Analysis at a professional level: a logistic regression model will be used to search for possible associations among the primary outcome variables and the secondary variables. First, each variable will be evaluated separately by univariate models and all the variables that are statistically significant or have a *P* ≤ .10 will be included in the multivariate model.

Analysis at a patient level: at this level hierarchal or multilevel methods will be used to take into account the structure of the data and to be able to introduce the variables of the patient in the estimation of the effect of the intervention. The levels will be professional (level 2) and patient (level 1). Multilevel logistic regression models will be made to estimate the effect of the intervention. The strategy of the analysis will be to estimate the effect of the intervention considering professionals as the random part. Afterwards, we will estimate the effect by introducing the variables of quality of care of the professional level (SQPP and SHCQ) and finally, we will study whether the introduction of patient variables modifies the effect of the intervention.

The level of significance will be set at 5% and the statistical package IBM SPSS Statistics v.23.0 for Windows will be used to perform the statistical analyses.

### 2.11 Ethics and dissemination

The study will be carried out following national and international norms (declaration of Helsinki) related to ethical aspects. This study protocol (version 1, November 15, 2016) was approved by the Ethical Committee of Clinical Investigation of the *Institut Universitari d’Investigació en Atenció Primària (IDIAP) Jordi Gol*, on March 15, 2017 (code P17/091). In addition, the study has been authorized by the Direcció Assistencial d’Atenció Primària of the ICS. The data included in the SIDIAP database will be anonymized and will be identified by an internal code, making subject identification impossible even by the investigative team, thereby guaranteeing the confidentiality of the data of the study participants included in the study according to the organic law on the protection of personal data (15/1999 of 13 December, LOPD). As the study is based on the analysis of an anonymized database, informed consent will not be collected from the patients. Any alteration of the study protocol will be submitted to the ethical committee of the IDIAP Jordi Gol for approval and will be published in clinical trials.gov.

The results of the study will be disseminated through informative meetings addressed to professionals of the ICS, communications to congresses, publications in scientific journals, and in the clinical trials.gov webpage.

### 2.12 Safety

The CDS-NVAF may influence clinical decision-making and may contribute in improving the adequacy of anticoagulant treatment in patients treated with VKAs. Changes in treatments are expected (from VKAs to DOACs), which would induce a decrease in the incidence rates of thromboembolic and hemorrhagic events in the medium and long term. Nonetheless, the monitoring of possible adverse effects will be carried out by the comparison of the incidence rates of the studied events between the 2 groups (intervention and control), monthly during the period of the intervention. If an increase in the incidence rates in the intervention group is detected at anytime, the CDS-NVAF will be deactivated by the principal investigator of the project. A data monitoring committee will not be established, as the evaluation of the adverse effects will be carried out by the SISAP in coordination with the principal investigator of the project.

## 3 Discussion

This is the first study to provide family physicians in primary care in Catalunya with a clinical decision support tool (CDS-NVAF) to improve the adequacy of anticoagulant treatment in patients with NVAF. The implementation of CDS-NVAF and its posterior evaluation is currently considered to be a strategy of priority by the ICS to improve the management of patients with NVAF and subsequently reduce thromboembolic and hemorrhagic complications and death derived from poor control of VKA anticoagulation.

The present study may have a positive immediate impact on clinical decision-making at the primary care level, with CDS-NVAD influencing the daily clinical practice of the general practitioners in ICS. On confirming the good results expected, the use of the CDS-NVAF can be extended to all ICS patients diagnosed with NVAF in Catalunya.

The study has received financial support to evaluate the results of 2 years after the beginning of the intervention, however, taking into account the importance of obtaining more knowledge on the impact of healthcare results in the mid or long term, the investigative team is seeking financial support to assess the impact of CDS-NVAF at 5 years postintervention.

The results obtained in the study may be of interest to all of Spain as well as other countries with similar healthcare models which use support tools for clinical decision-making.

With regard to the limitations of the study, the SIDIAP database includes secondary data, and thus, there may be a certain level of underregistration of variables of interest, although this should not differ between the 2 study groups. The quality of the registry is guaranteed in that most of the study variables make up part of different indicators of quality (SHCQ and SQPP) which are incentivated in the payment systems for objectives of the ICS. There is no problem in the registry of the treatment variables as the billing system of the pharmacy offices collects these data. Another limitation is that the study is unblinded for physicians and there may be contamination between groups which may limit the conclusions of the study. Nonetheless, we aim to evaluate the effectiveness of the intervention in the real-life setting related to the usual clinical practice in which this type of situation is frequent. The investigative team believes that randomization by sectors and not by physicians will reduce the probability of contamination between groups. Another limitation of the study is that the tool provides information about the adequacy of the VKA but not the adequacy of the DOACs, which should be taken into account in result interpretation.
