# A new clinical decision support tool for improving the adequacy of anticoagulant therapy and reducing the incidence of stroke in nonvalvular atrial fibrillation: A randomized clinical trial in primary care: Erratum

**DOI:** 10.1097/MD.0000000000009915

**Published:** 2018-02-09

In the article, “A new clinical decision support tool for improving the adequacy of anticoagulant therapy and reducing the incidence of stroke in nonvalvular atrial fibrillation: A randomized clinical trial in primary care”,^[1]^ which appeared in Volume 97, Issue 3 of *Medicine*, the following funding information should appear:

The project was financed in 2016 by the Departament de Salut of the Generalitat de Catalunya (contact information: Travessera de les Corts, 131, 08028 Barcelona), through an external competitive call (Pla estratègic de recerca i innovació en salut (PERIS) - Subvencions per a projectes de recerca orientats a l’atenció primària), grant number SLT002/16/00146. The funder financed the project but will not participate in any phase of the investigation. The funder will not have any authority over any of the activities of the project.
